# Evaluation of seasonal changes of serum and plasma estradiol-17β, progesterone and testosterone in dolphins (*Tursiops truncatus*) by chemiluminescence

**DOI:** 10.14202/vetworld.2015.977-982

**Published:** 2015-08-12

**Authors:** Santo Fragalà, Pietro Medica, Francesco Grande, Irene Vazzana, Esterina Fazio

**Affiliations:** 1 Department of Veterinary Sciences, University of Messina, 98166 Messina, Italy; 2 Zoomarine Acquatic Park, Torvaianica, Roma, Italy; 3 Istituto Zooprofilattico Sperimentale della Sicilia “A. Mirri”, Via G. Marinuzzi 3, 90129 Palermo, Italy

**Keywords:** dolphin, estradiol-17β, progesterone, testosterone

## Abstract

**Aim:**

The purpose of the research was to test and validate an innovative and safe chemiluminescence method to evaluate sexual hormones in serum and plasma samples of *Tursiops truncatus*.

**Materials and Methods:**

The research was performed on 9 bottlenose dolphins entertained in Oltremare and in Zoomarine aquatic parks, sampled by the tail vein or from the ventral one and an ultrasound monitoring, throughout a 6 months period. Blood samples were analyzed using a chemiluminescence method. Data obtained were compared to radioimmunoassay and enzyme immuno assay reference data, with the purpose to test and validate this method, through the calculation of the coefficient of variability, and its reliability on serum and plasma samples. A one-way analysis of variance was applied to test the effect of time on serum and plasma hormonal changes.

**Results:**

Mean concentrations of estradiol-17β in serum were equal to 149.07±6.82 pmol/L, and in plasma equal to 159.14±12.99 pmol/L; mean values of progesterone in serum were equal to 0.69±0.05 pmol/L, and in plasma equal to 0.64±0.05 pmol/L; mean values of testosterone in serum were equal to 44.43±14.42 nmol/L, and in plasma equal to 48.99±11.20 nmol/L.

**Conclusion:**

It would be interesting to widen the investigations on a larger number of subjects, in which the relationship between the concentrations of free and binding steroid hormones, with the dosing of binding proteins, would define the physiological ranges of reference in the *T. truncatus*.

## Introduction

The steroid hormones arouse great interest in different zoological competences, among which the evolutionary physiology, ethology and biology of the preservation [1]. Environmental, nutritional or social influences may affect the timing of reproductive cycles of marine mammals [2,3]. Hence, it is impossible to separate a reproductive cycle from its context [4].

Monitoring the sexual cycle in captive animals represents an efficient tool to promote mating and to facilitate the assisted fertilization using specific protocols. In the past, measuring concentrations of reproductive steroids in the blood, salivary, ocular and vaginal secretions was the most common method of determining reproductive status of marine mammals, using radioimmunoassay (RIA) method and competitive enzyme immuno assay (EIA) technique [5,6]. Both methods are binding assays that depend on the progressive saturation of a specific antibody by a substance and the subsequent determination of bound and free phases using antibodies labeled with either a radioactive isotope (RIA) or an enzyme (EIA). Sometimes, RIA can produce misleading results, as they measure not only dimeric forms of a hormone but also the free subunits [7]. The continuing effort to improve their diagnostic values has produced a set of reference values for blood constituents, including steroid hormones [5,8-12].

On these scientific bases and in the presence of discordant data, it seemed interesting to provide a scientific contribution, addressed to the comparison of alternative methods to monitor the gonadic activity in *Tursiops truncatus*. The purpose of research was to test and validate an innovative and safe chemiluminescence method, to evaluate sexual hormones in serum and plasma samples of *T. truncatus*, by taking into account the seasonal modifications in according to the reproductive season, the age, and the affiliation to the different aquatic parks.

## Materials and Methods

### Ethical approval

All procedures described here were performed in accordance with the guidelines of the Italian Minister of Health for the care and use of animals (D.L. 4/3/2014 n. 26) and European Union (EU) (Directive 2010/63/EU) and the EAAM standard for establishments housing bottlenose dolphins (http://www.eaam.org/housing_standards/). Samples were collected under permits from the Zoomarine Acquatic Park and Oltremare Riccione Park. All methods and procedures were under approval by the Messina University Institutional Board for the Care and Use of Animals and were in compliance with the guidelines.

### Animals and environmental conditions

The research was carried out on 9 bottlenose dolphins (*T. truncates)*, which 5 no pregnant mature females and 4 mature males, of different age, belonging to different social hierarchies housed in two Italian facilities (Zoomarine, Rome; Oltremare, Riccione, Italy).

The aquatic parks of Zoomarine entertained 5 dolphins (2 females and 3 males), and of Oltremare of Riccione entertained 4 dolphins (1 males and 3 females), respectively. The age of the subjects was ranging between 10 and 47 years.

The Zoomarine aquatic park entertained the animals in an island, constituted by 5 tubs, respectively a show tub, a maternity, two accessories and a therapeutics-veterinary, for a total volume of 7.8 million liters of water. The maximum depth was of 6 m, reached in the show tub. The disinfection happened using sodium hypochlorite and the optimal salinity that varied between 24% and 27%. The filtering was mechanic through sand filters, by infinite loop, of around 2 h, and the cleaning of the tubs and the filters were performed, in according to necessity. The optimal pH, so that the kinetics of the dissociation of the chlorine in watery environment was constant and was obtained through the addition of hydrochloric acid, with the use of special pumps, with inclusive oscillations between 7.2 and 7.6. The temperature ranged between 14°C and 29°C. The chemical values, as free chlorine, total chlorine, combined chlorine, salinity, pH/redox values and water temperature were constantly monitored. In the same way, ammonia, nitrates, phosphates and aluminum were monitored once a week. The bacteriological values, like total fecal coliforms (*Escherichia coli*), *Pseudomonas* spp., *Escherichia* strains, were monitored once a month.

The entertained animals were five examples of *T. truncatus*, 2 mature no pregnant females and 3 mature males, fed more times a day, during the sessions of training, with fish and squids, in the quantities established by the veterinary team and with the zoological manager, in according to the kilocalories calculation, the demands of the biometric seasonal and physiological data of each animal. The feeding founded on the administration of *Loligo vulgaris* (squid), *Micromesitius poutassou* (melu), *Trachurus trachurus* (suro), *Sprattus sprattus* (sprat), *Clupea harengus* (herring), *Mallotus villosus* (capelin), *Osmerus eperlanus* (sparling), *Scomber scombrus* (mackerel), and multivitaminics complex were added.

Oltremare aquatic park entertains 6 tursiops, 4 females, 2 males and 1 grampus. The researches were carried out only on 4 of the tursiops, 3 mature no pregnant females and 1 mature male. The animals entertained in a lagoon, that includes the show tub, the medical and reproduction tubs, with mobile fund, for a total volume of 8 million liters of water. The filtration system was to infinite loop, and it was performed by sand filters, ozone, degassing towers and protein skimmer, used for the separation of the organic residues. The disinfection was made by the addition of sodium hypochlorite. Water was regularly changed by 10% monthly and the cleaning of the tubs was performed every 5 days. Salinity was guaranteed by the introduction of brine in the tub that was mixed to the plain water of the changes. The pH values were constantly maintained, using the hydrochloric acid, at a value of 7.7. The temperature ranged between 16°C and 26°C.

The animals were fed, more times a day, during the sessions of training, with herrings, squids, capeling and sprat (the correspondent scientific name was above indicated). The alimentary quantity was established by the veterinary and training staff in according to season, the activity and biometric measurements that were weekly performed.

### Blood sampling

All the subjects were submitted to blood sampling every 4 weeks, from April to September, throughout a 6 months period, to compare the seasonal concentrations of serum and plasma sexual hormones. All the samplings were performed in optimal conditions of extreme calm and tranquility, since the subjects were totally accustomed to the operator.

Blood samples were obtained from the dorsal surface of the fluke or from the ventral one, presented voluntarily and held in the appropriate position until sampling was completed as signaled by the trainer, and based on the modus operandi of the veterinary staff, only during routine physical examination of the animals. Total sampling was carried out between 09:00 and 12:00 a.m. At this purpose, green butterflies were used for vacuum tubes (Terumo MN-SV 21).

Blood samples were picked in test-tubes with ethylenediaminetetraacetic acid (EDTA). Plasma and serum samples, at least 1.5 ml for each sample, were centrifuged at 2500 rpm, at a temperature between 20°C and 30°C and, finally, preserved in Eppendorf and stored at −20°C until assayed. A total number of steroid hormone determinations were 45 for plasma and 45 for serum.

### Chemiluminescence assays

Total sexual hormone assays were performed using immulite 2000, and the samples were processed in duplicate.

For estradiol-17β (E_2_) assay was used a chemical luminescent enzyme in the solid phase (immulite 2000 estradiol immuno-assay). The solid phase was composed with a polyclonal anti-estradiol antibody (rabbit’s antibody). The reagent contained alkaline phosphatase, conjugated with estradiol. The ­conjugated estradiol-enzymatic competed with the estradiol in the sample for limited sites bounded to antibodies on the solid phase. The sample and the reagent in excess were removed by centrifugal washing. Finally, the substratum’s chemiluminescence was added to the solid phase and a signal in proportion to the binding enzyme was generated. The volume required for every cycle of incubation (1 × 60 min) was of 25 µl of serum or plasma. It was recommended to redose after dilution the samples with superior values of 1200 µl/ml. The precision was valued by measuring the repetition and reproducible, with a coefficient of variability (CV) of 6.3% intra-assay and 9.3% inter-assay, respectively.

For progesterone (P_4_) assay a sequential competitive immunoassay (immulite 2000 progesterone) was used. The volume required for every cycle of incubation (2 × 30 min) was of 50 µl of serum or plasma. All samples that had superior levels compared to calibration range test were diluted before the test, and the corrections related to dilutions were manually effected. Nevertheless, data obtained using plasma were interpreted with prudence, considering that the EDTA had a meaningful effect on the results. The CV was of 6.3% intra-assay and of 7.9%, inter-assay, respectively.

For testosterone (T) assay a chemical luminescent enzyme of a solid phase (immulite 2000 total testosterone) was used. For every cycle of incubation (1 × 60 min), 20 µl of serum or plasma were required. Nevertheless, data obtained using plasma were interpreted with prudence, considering that the EDTA has a meaningful effect on the results.

### Statistical analysis

The statistical analysis was carried out by one-way analysis of variance (ANOVA) for repeated measures (RM ANOVA) to appraise the effect of time on the plasma and serum hormonal changes. When the value of F was meaningful, to compare the differences between the mean values of the plasma and serum concentrations in the different times of observation, a *post-hoc* Bonferroni’s multiple comparison tests was used. To compare the monthly differences of plasma and serum hormonal concentrations a paired *t*-test was applied. The level of significance was set at p<0.05.

The calculations were performed with the Software PRISM (GraphPad Software Inc., San Diego, CA, USA).

## Results

Total mean plasma and serum E_2_ concentrations were equal to 159.14±12.99 (pmol/L) and to 149.07±6.82 (pmol/L), respectively.

**Figure-1:**
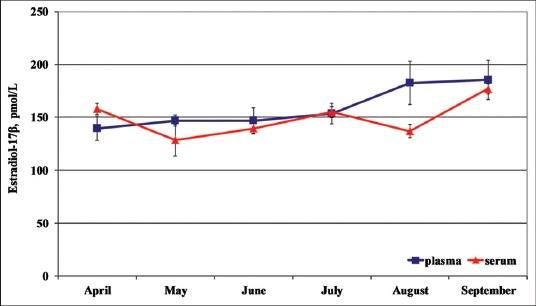
Seasonal plasma and serum (mean ± standard deviation) estradiol-17β in dolphins.

Concerning the seasonal concentrations (Figure-1) observed from April to September, the lowest plasma E_2_ concentrations were observed in April (139.48±11.60 pmol/L) and the highest in September (185.38±18.62 pmol/L). In addition, the lowest serum E_2_ concentrations were observed in May (128.33±14.71 pmol/L) and the highest in September (176.94±4.63 pmol/L).

The individual seasonal plasma E_2_ pattern was more homogeneous and superimposable than serum one. Moreover, no significant differences between plasma and serum E_2_ concentrations were observed, in according to the different affiliation to the two different aquatic parks and different age; nevertheless, Oltremare’s older specimens (20-47 years old) showed higher E_2_ concentrations than Zoomarine’s younger specimens (10-20 years old). One-way RM ANOVA did not show any effect of time on the E_2_ changes.

**Figure-2:**
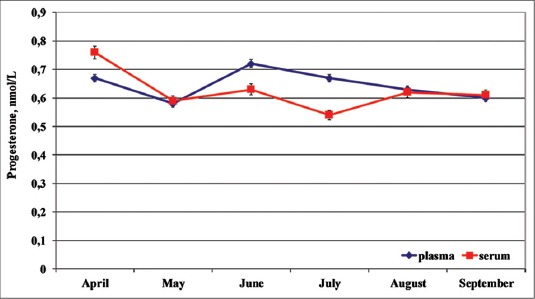
Seasonal plasma and serum (mean ± standard deviation) progesterone in dolphins

Total mean plasma and serum Progesterone concentrations were equal to 0.64±0.05 (nmol/L) and 0.62±0.05 (nmol/L), respectively. Concerning the seasonal changes (Figure-2), the lowest plasmatic P_4_ concentrations were observed in May (0.58±0.05 nmol/L) and the highest in June (0.72 ± 0.03 nmol/L). The lowest serum P_4_ concentrations were observed in July (0.54±0.06 nmol/L) and the highest in April (0.76±0.08 nmol/L).

The individual seasonal plasma and serum P_4_ pattern was homogeneous and superimposable. No significant differences between plasma and serum P_4_ concentrations were observed, in according to the different affiliation to the two different aquatic parks and different age; nevertheless, Oltremare’s older specimens (20-47 years old) showed higher P_4_ concentrations than Zoomarine’s younger specimens (10-20 years old). One-way RM ANOVA did not show any effect of the time on the P_4_ changes.

The ultrasound scan confirmed a physiological seasonal cyclicity with the presence of growing follicles.

**Figure-3:**
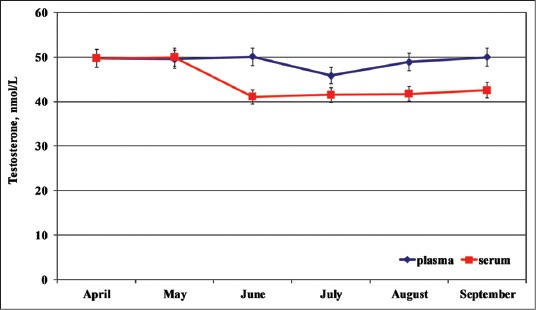
Seasonal plasma and serum (mean ± standard deviation) testosterone in dolphins.

Total mean plasma and serum Testosterone concentrations were equal to 48.99±11.20 (nmol/L) and 44.43±14.42 (nmol/L), respectively. Concerning the seasonal changes (Figure-3) the lowest plasmatic T concentrations were observed in July (45.83±19.28 nmol/L) and the highest in June (50.09±10.75 nmol/L). The lowest serum T concentrations were observed in June (41.05±16.78 nmol/L) and the highest in May (50.00±10.92 nmol/L).

The individual seasonal plasma T pattern was more homogeneous and superimposable than serum one. Moreover, no significant differences between plasma and serum T concentrations were observed, in according to the different affiliation to the two different aquatic parks and different age. One-way RM ANOVA did not show any effect of the time on the T changes.

## Discussion

The results obtained showed that plasma and serum estradiol-17β, progesterone and testosterone concentrations substantially ranged within the physiological ranges reported in literature for Dolphins and Whales, under different physiological conditions, although the different assay techniques made difficult, in some cases, the comparison [5,8,9].

In addition, considering that the dolphins sampled in this study were trained to allow blood sampling and to voluntarily offer their tail flukes, the steroid hormone concentrations obtained from these specimens could be considered as resting, non-stressed sexual hormone values.

The results showed the peculiar tendency of *T. truncatus* to maintain almost superimposed estradiol-17β and progesterone concentrations, both in plasma and in serum samples.

Seasonal variations were observed in circulating plasma and serum E_2_ concentrations that peaked in September and decreased during April and May, respectively.

On the other hand, the comparison of the highest plasma and serum E_2_ concentrations of females in September could be explained on the basis of the period of greatest sperm production and density of males that were coincident with the period of breeding activity, as reported by Schroeder and Keller [9]. Nevertheless, it is possible to suppose that their presumed hierarchical position had any effects on the highest E_2_ concentration, observed in September. In fact, it is possible that the dominant subjects induced a positive feedback on the release of gonadotropins, with a permissive effect on the synthesis and secretion of estrogens, with the best reproductive performance, as, besides, already reported in other species [13].

In addition, the lowest E_2_ concentrations observed between April and May confirmed partially the seasonal cyclicity reported in spring-summer and autumn for tursiops [14]. Nevertheless, the continuous secretion of E_2_ from April to September indicates that folliculogenesis was not arrested, as was in seasonal breeders of domestic animals. This assumption confirms that most cetaceans, including dolphins, are spontaneous ovulators and seasonal breeders and that *T. truncatus* may ovulate several time (poly-oestrus cycles) [4]. Furthermore, higher standard deviation of plasma E_2_ concentrations, rather than serum sampling, confirmed the preferential use of serum for the chemiluminescence assay of this hormone.

Regarding the circulating P_4_ changes, although the plasma concentrations are lower than those reported by Atkinson *et al*. [15] in *Pseudoorca crassidens*, they are in substantial agreement with the one reported by Walker [16] in *Orcinus orchus*. Nevertheless, the plasma P_4_ concentrations reported by Atkinson *et al*. [15] in adult female killer Whales were already relatively high when compared to ranges reported in the literature for dolphins and killer whales [17-19]. Seasonal plasma and serum P_4_ concentrations peaked in June and decreased during July and September. The higher P_4_ levels of Oltremare’s subjects than those one of Zoomarine confirmed an effect of age [4,15].

The homogeneity of the P_4_ concentrations, the existence of a contained standard deviation and the overlap of the plasma and serum trend show that the biological samples of plasma and serum could be used for chemiluminescence assay.

Seasonal serum and plasma T concentrations peaked in May and June, respectively, and decreased during July and August, as reported by Schroeder and Keller [9]. The overlap, nearly total, of the circulating T concentrations, were unknown, but it could be suggested a different equilibrium in the kinetics of this hormone metabolism in these dolphins, in according to the social grouping. Thus, the high variability of T of these specimens does not necessarily imply an elevated metabolic state but it signals instead a high plasma-binding capacity for this hormone, as reported in previous study for thyroid and adrenal hormones of dolphin [20]. In addition, recent observations reported an ability of the dominant male cetacean populations to exert a suppressive effect on reproductive function in the subordinate animal, as reported in some terrestrial species [1].

Regarding the effect of the circadian rhythm on the hormonal changes, we feel to exclude, with enough certainty, such probability, having effected the samplings at the same time, both for the Oltremare’s and Zoomarine’s samples, despite the existence of a circadian rhythm described for the steroid hormones in *T. truncatus* and *Orcinus orca*, with the highest levels in the 1^st^ h of the morning and more lower part of the afternoon, together to annual modifications [21,22].

Estrogen and progesterone concentrations fluctuate markedly on a day to day basis during ovulation and would have to be monitored serially, over several days to distinguish transient changes associated with the physiological state [20]. Thus, it appears that our blood samplings were sufficient to bias the baseline chemiluminescence values of reproductive hormones for these dolphins.

In addition, the quality of the water and the feeding in both aquaria were similar as the same company administered them; hence, it possible to exclude an influence of these variables on hormonal changes.

A final consideration, even if not less important, was mandatory regarding the circulating total, free or binding steroid hormones from and of the relative tissue sensitivity. Our study did not include an analysis of binding globulins to elucidate their relationship to reproductive hormones.

Different studies have appraised the ability of the relative binding globulins and calculated the concentrations of free steroids [23]. Since only the free steroids was generally considered available for the diffusion in the tissue, different researchers have supported the hypothesis that the concentrations of free steroids are biologically more active compared to the concentrations of total steroids [23,24]. The concentrations of the binding globulins of the steroid hormones vary seasonally too, and they were regulated, for instance, by the concentrations of testosterone [25,26]. Besides, if the concentrations of total and free steroids are compared, their seasonal rhythm is often modified, or it entirely disappears. Hence, the seasonal changes of binding globulins can influence, therefore, the behavioral and physiological effects promoted by the steroids hormones, (i.e. testosterone) [11,26].

Beyond their diagnostic values, previous studies mainly focused on the biological and methodological sources of variance of the sexual hormones, founding that detailed knowledge of the state variables, associated with inter- and intra-individual variability, is an essential prerequisite to reducing the contrasting literature in this field.

In so far, within the future perspectives, it would be interesting to widen the investigations on a larger number of subjects, in which the relationship between the concentrations of free and binding steroid hormones, with the dosing of binding proteins, would define the physiological ranges of reference in the *T. truncatus*.

The combination of this technique with clinical and behavioral observations could allow objective evaluation of the annual or seasonal pattern of reproductive activity.

## Authors’ Contributions

All Authors have made substantial contributions to each step of experimental procedure and manuscript preparation. The idea for the paper was conceived by SF and EF. The experiments were performed by SF and FG. The laboratory analysis and data were analyzed by IV and PM, respectively. The paper was written by EF. All authors read and approved the final manuscript.
